# Solitary infantile myofibromatosis of the petrous bone: a diagnostic pitfall in uncommon location illustrated by a case report

**DOI:** 10.1093/jscr/rjad237

**Published:** 2023-05-12

**Authors:** Hafsa El Ouazzani, Imane Azzam, Zainab Benyahya, Rachida Chehrastane, Abdelilah Oujilal, Fouad Zouaidia, Nadia Cherradi

**Affiliations:** Department of Pathology HSR, Ibn Sina University Hospital Center, Rabat, Morocco; Mohammed V University in Rabat, Morocco; Mohammed V University in Rabat, Morocco; Department of Oto-Rhino-Laryngology HSR, Ibn Sina University Hospital Center, Rabat, Morocco; Mohammed V University in Rabat, Morocco; Department of Oto-Rhino-Laryngology HSR, Ibn Sina University Hospital Center, Rabat, Morocco; Mohammed V University in Rabat, Morocco; Department of Pediatric Radiology, Ibn Sina University Hospital Center, Rabat, Morocco; Mohammed V University in Rabat, Morocco; Department of Oto-Rhino-Laryngology HSR, Ibn Sina University Hospital Center, Rabat, Morocco; Mohammed V University in Rabat, Morocco; Department of Pathology Ibn Sina, Ibn Sina University Hospital Center, Rabat, Morocco; Department of Pathology HSR, Ibn Sina University Hospital Center, Rabat, Morocco; Mohammed V University in Rabat, Morocco

**Keywords:** Infantile myofibromatosis, Solitary, Petrous bone, Histopathology

## Abstract

Infantile myofibromatosis (IM) is the most common fibrous disorder of infancy and early childhood. Solitary intracranial involvement is rare and often unrecognized. This makes its early diagnosis and adequate management difficult. The majority of lesions are localized to the skull or dura with variable intracranial extension. Herein, we report a misdiagnosed and aggressive presentation of a solitary IM of the petrous bone. Our aim is to discuss histopathological differential diagnoses and management difficulties.

## INTRODUCTION

Infantile myofibromatosis (IM) is a distinct clinical disorder of infancy and early childhood characterized by solitary, multicentric without visceral involvement and multicentric IM with visceral compromise or generalized neoplasms [1].

Solitary lesion in craniofacial bone is rare. In our knowledge, initial petrous bone involvement has not been reported in literature [2, 3]. In such location, a wide morphologic spectrum is encountered. This complicates the diagnosis and delays the therapeutic management.

Herein, we report an illustrative case of IM of the right petrous bone and mastoid portion of temporal bone, extending to parotid region and brain in 5-year-old girl.

## CASE

We report the case of 5-year-old girl without any significant previous medical history. She suffered of persistent right otorrhea associated with a banal polyp of the external auditory meatus. Initial computed tomography (CT) scan revealed congestion of the middle ear cavities with osteolysis of the petrous bone. The biopsy was in the favor of inflammatory changes. The infectious origin has been evoked.

**Figure 1:**
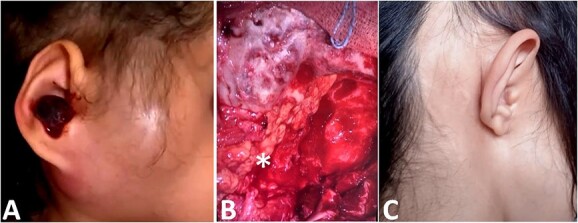
(**A**) Image showing a right retro-auricular and parotid swilling with a polyp of the external auditory meatus. (**B**) Reconstruction with abdominal fat (star). (**C**) Clinical picture after surgery and reconstruction.

**Figure 2:**
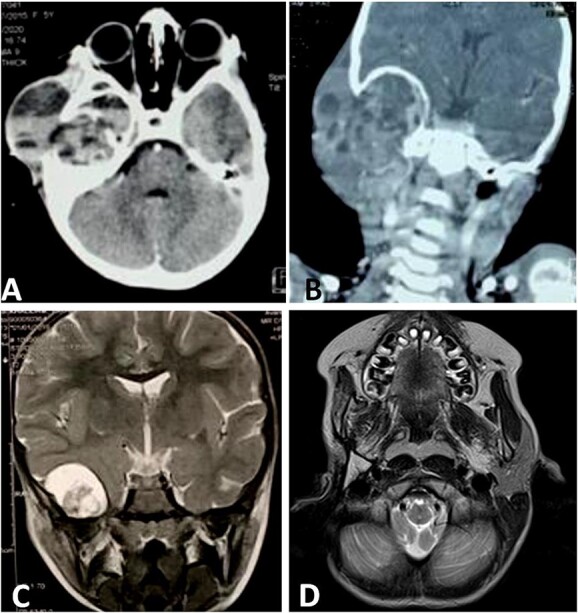
CT scan shows a cystic process of the middle ear cavities with a significant osteolysis of the petrous bone, mastoid and temporal bone (**A**) with extension to parotid region (**B**) and homolateral meninge and brain (**C**). Follow-up MRI shows no sign of recurrences (**D**).

We noted an aggravation of symptoms with malignant otitis, progressive facial paralysis, retro-auricular swilling (Fig. 1A) and appearance of neurological signs. The CT scan revealed a significant osteolysis of the petrous bone, mastoid and temporal bone with homolateral meningeal and cerebral extension. This suggested a malignant process (Fig. 2).

**Figure 3:**
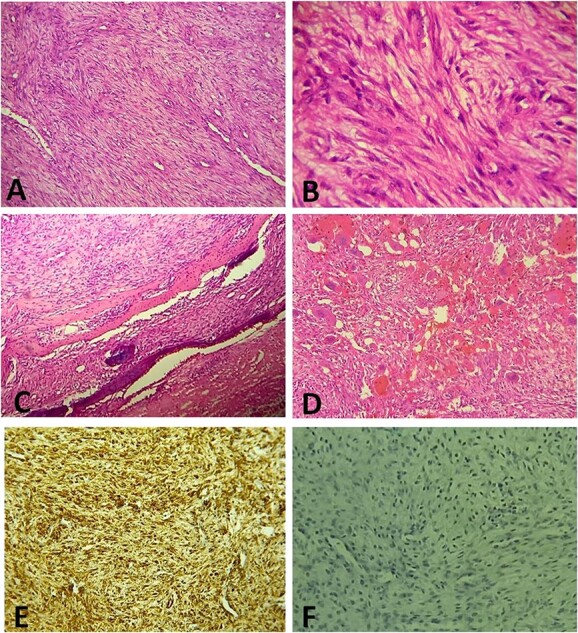
(**A**) Histologically, the tumor is composed of spindle cell proliferation with an alternation of hypocellular zones and cellular zones associated with haemangiopericytic architecture (Hematoxylin-Eosinx10). (**B**) Tumor cells have elongated nuclei with fine chromatin and a small nucleolus (Hematoxylin-Eosinx40). (**C**) Tumor infiltrating temporal bone (Hematoxylin-Eosinx20). (**D**) Note the polymorphic inflammatory reaction rich in giant cells (Hematoxylin-Eosinx20). At the immunohistochemistry, tumor cells are positive for SMA (**E**) and negative for Desmin, Myogenin, CD34 and ALK (**F**).

A surgical biopsy was performed and an immunohistochemistry panel was requested. Histologically, the tumor was composed of spindle cell proliferation with an alternation of hypocellular zones and cellular zones associated with haemangiopericytic and nodular architecture. Tumor cells have elongated or round nuclei with fine chromatin and small nucleolus. Mitosis figures are rare. Polymorphic inflammatory reaction rich in giant cells was observed (Fig. 3). At the immunohistochemistry, tumor cells were positive for smooth muscle actin (SMA) and negative for Desmin, Myogenin, CD34 and anaplastic lymphoma kinase ALK (Fig. 3E and F). The definitive diagnosis was an infantile intracranial myofibromatosis.

Then, the patient underwent total surgical resection preceded by chemotherapy. At surgery, the tumor was noted to be located in the petrous bone itself and did not arise from the underlying dura. Therefore, the underlying dura mater was preserved. The reconstruction was made by filling with abdominal fat and biological glue, with a good result (Fig. 1B and C). The follow-up magnetic resonance imaging (MRI) showed no sign of recurrences with 3 years survival (Fig. 2D).

## DISCUSSION

Infantile myofibromatosis (IM)/myofibromas were first described by Stout in 1954 as ‘congenital generalized fibromatosis’ [4]. IM is now considered within the spectrum of tumors with perivascular myoid differentiation based on the involvement of myopericytoma located in the vessel wall [5]. Recent studies suggest familial autosomal-dominant inheritance due to a mutation in PDGFRB and NOTCH3 genes [4].

Many affected patients will present for care to an otolaryngologist–head and neck surgeon because about one-third of tumors arise in the head and neck [6].

Bones of skull involvement is rarely observed in the solitary form; in our knowledge, initial petrous bone involvement has not been reported in the literature [7].

The clinical and radiological management of these lesions has yet to be well defined, because of the variability of clinical and radiological presentation and the histological difficulties with diagnosis [1].

Histologically, the tumor shows well-circumscribed tapered cell lobes resembling smooth muscle cells. At its center, perivascular round cells (hemangiopericytomatous pattern) are usually observed giving a biphasic appearance [8]. Histological features mimicking malignancy, such as local invasion, increased cellularity, rich vascularity and extensive necrosis, are not uncommon. In such case, pathological distinction between myofibromatosis, desmoid fibromatosis, infantile fibrosarcoma or any other sarcomas with myopericytic characteristics can be challenging [4]. Ancillary techniques (immunohistochemistry and molecular genetic testing) may be of help. Regarding immunohistochemistry, IM express both vimentin and SMA. Beta-catenin and striated muscles markers (Desmin and Myogenin) are usually negative [9].

Unlike IM, desmoid fibromatosis rarely affects infants and has an aggressive biological behavior. It is composed of collagenous fibers with long, sweeping fascicles, thin-walled vessels, bland cells with mild to moderate cellularity and minimal atypia. Tumor cells are SMA positive and nuclear beta-catenin positive. CTNNB1 and APC mutations may be seen.

As for fibrosarcoma, it is a malignant tumor with poor prognosis characterized by the wheel-like shape of tumor cells in fascicles or herringbone pattern displaying nuclear atypia [6]. ETV6–NTRK3 gene fusion transcript has been seen at 70% [9].

IM may show extensive hyalinization or other regressive features including hemorrhage, cystic degeneration, calcification and inflammatory infiltrate. These changes can mask the tumor proliferation and mimic an inflammatory or infectious process. As in our case, the cystic and fluid lysis of the bone was in favor of otitis. The tumor was misdiagnosed and it progressed to facial paralysis and cerebral compression.

Some authors report that these lesions tend to be locally aggressive, with infiltrative growth and a strong tendency to recur after excision, whereas other reports suggest a benign character with spontaneous regression [1]. The underlying mechanism of tumor regression and growth remains unknown, but it has been suggested to be related to angiogenic stimulation and regression, both triggered by basic fibroblast growth factor [4]. 

A wait-and-see strategy is appropriate for some patients. If resection without squeal appears feasible, solitary lesions may be removed surgically. Systemic therapy is recommended in case of life-threatening progressive disease; typically, due to compression of vital disease. Alternatively, a few children with PDGFRB-mutation IM have been treated successfully with imatinib and sunitinib [10].

## CONCLUSION

Solitary intracranial IM is rare and carries a worse prognosis in this localization than in others. A misdiagnosed of more aggressive tumors such as fibrosarcoma should be avoided to properly adapt the management.

## DATA AVAIBILITY

No new data were generated or analysed in support of this research.
